# Moral Distress and Occupational Burnout in US Physicians

**DOI:** 10.1001/jamanetworkopen.2026.3161

**Published:** 2026-03-24

**Authors:** Michael A. Tutty, Colin P. West, Liselotte N. Dyrbye, Hanhan Wang, Lindsey E. Carlasare, Christine A. Sinsky, Mickey Trockel, Tait D. Shanafelt

**Affiliations:** 1 American Medical Association, Chicago, Illinois; 2 Mayo Clinic, Rochester, Minnesota; 3 University of Colorado School of Medicine, Denver; 4 Stanford University, Palo Alto, California; 5 Merrimac, Wisconsin

## Abstract

**Importance:**

Exploring the relationship between moral distress and occupational burnout is necessary to understand the association between these constructs.

**Objective:**

To evaluate moral distress among physicians and US workers, and to explore the association of moral distress with burnout, intent to leave (ITL) current position, and intent to reduce clinical work hours (ITR).

**Design, Setting, and Participants:**

This cross-sectional national survey study included physicians from all specialties and a probability-based sample of employed nonphysicians. Participants were aged 29 to 65 years. Data were collected between October 19, 2023, and March 5, 2024. Data were analyzed from June 30 to October 20, 2025.

**Main Outcomes and Measures:**

Moral distress was measured using the Moral Distress Thermometer (MDT, range 0-10), with a high level of moral distress defined by a score of 4 or higher. Burnout was measured using the complete Maslach Burnout Inventory (MBI). Professional fulfillment was measured using the Stanford Professional Fulfillment Index. ITL and ITR were measured using a standardized item with response options of none, slight, moderate, likely, and definitely.

**Results:**

This survey study included 5741 physicians and 3501 nonphysician US workers. The median (IQR) age of physicians was 53 (44-62), and included 3262 men (58.0%), 2255 women (40.1%), and 107 individuals who responded other (1.9%). The mean (SD) moral distress score for physicians was 3.29 (2.81), with 2243 (39.1%) reporting a high level of moral distress (4 or more considered high). On multivariable analysis, women physicians had higher odds of moral distress (OR, 1.29; 95% CI, 1.12-1.48). Compared with internal medicine subspecialists, emergency medicine physicians (OR, 3.16; 95% CI, 2.27-4.4) and general internal medicine physicians (OR, 1.92; 95% CI, 1.42-2.59) were more likely to report high levels of moral distress. Mean emotional exhaustion and depersonalization scores, as well as the proportion of physicians with burnout, were higher with each 1-point increase in moral distress score. The overall correlation between the emotional exhaustion score and moral distress score was *R* = 0.55 (*P* < .001) while the correlation between the depersonalization score and moral distress score was *R* = 0.50 (*P* < .001). Additionally, 1068 of 3477 physicians (30.7%) with a moral distress score less than 4 had burnout symptoms compared with 1675 of 2231 physicians (75.1%) with scores of 4 or more (*P* < .001). The prevalence of ITL and ITR was higher for each 1-point increase in moral distress score. For example, 619 of 3404 physicians (18.2%) with low moral distress reported ITL within 24 months compared with 748 of 2171 (34.5%) among those with high moral distress (*P* < .001). Compared with other US workers, physicians had markedly higher odds of experiencing moral distress (OR, 4.40; 95% CI, 3.84-5.06).

**Conclusion and Relevance:**

In this survey study, moral distress was common among physicians and experienced at higher rates than the general US working population. Understanding the differences between moral distress and burnout may allow organizations to more effectively implement interventions to address both concerns among clinicians.

## Key Points

**Question:**

What is the level of moral distress and the association between burnout, intent to leave (ITL), and intent to reduce work hours (ITR) among physicians and US workers?

**Findings:**

In this survey study of 5741 physicians and 3501 US workers, the mean moral distress score for physicians was 3.29, with 39.1% reporting a high level of moral distress; physicians had higher odds of experiencing moral distress than other US workers. The prevalence of burnout, ITL, and ITR was higher for each 1-point increase in moral distress score among physicians.

**Meaning:**

These findings suggest that moral distress is common among physicians and experienced at higher rates than the general US working population.

## Teaser

This survey study evaluates moral distress among physicians and US workers and examines the association between moral distress, burnout, intent to leave, and intent to reduce clinical hours.

## Introduction

In its ideal form, the practice of medicine is a fundamentally moral activity in which physicians act in their patients’ best interests.^1^ However, physicians frequently encounter situations in which upholding professional ethical values is challenged when they feel their environment constrains the ethically right course of action.^2^ For example, a physician who feels forced to continue artificial life support in a medically futile situation or has other concerns about the quality of care may experience moral distress.^3^ When the experience of moral distress is sustained or occurs frequently, it can progress to moral injury, characterized by impaired function, a sense of betrayal, and feelings of guilt, shame, and anger.^4,5^

Previous studies have identified moral distress in medical students, residents, and practicing physicians.^6,7,8^ Challenging clinical circumstances, ethical dilemmas, problems with communication and teamwork, and reimbursement structures that create conflicts of interest can all lead to moral distress.^9^ Recent legislative and judicial decisions impacting reproductive care and gender affirming care have also increased moral distress.^10,11,12,13,14^ Decisions about end-of-life care have been reported to be a frequent source of moral distress.^15,16,17^

Moral distress can also contribute to occupational burnout. Research addressing physician burnout and job satisfaction highlights that the changing and increasingly complex US health care system has placed considerable burdens on physicians.^18,19^ Burnout in physicians has critical implications for the health care delivery system, with evidence indicating it decreases quality of care, erodes patient experience, increases the cost of care delivered, and is associated with intent to reduce the number of clinical hours worked each week (ITR) and increased intent to leave clinical practice altogether (ITL), which reduces access to care.^20,21,22,23,24,25,26,27,28,29,30,31^

The theoretical constructs and available data suggest that burnout and moral distress are related but distinct experiences.^4,32^ Both are associated with consequential outcomes for physicians, but how they overlap and differ is not well understood. Burnout is brought on by prolonged workplace stress and is characterized by emotional exhaustion (EE), depersonalization (DP), and a reduced sense of personal accomplishment.^33^ For physicians, long hours, heavy workloads, loss of flexibility and control, inefficiency in the practice environment, poor teamwork, and a professional culture of perfectionism and low self-valuation can lead to burnout.^34,35,36,37,38,39,40^ Moral distress can be caused by clinical situations (eg, treatment decisions), personal factors (eg, perceived powerlessness), and external factors (eg, institutional policies).^41^ While many situations are likely to cause moral distress for most physicians, moral distress is an individual experience, and some situations may cause moral distress for 1 person but not another based on an individual’s experience, training, and professional beliefs.^3,42,43^ Understanding the differences between moral distress and burnout may allow organizations to more effectively implement interventions to address these concerns among clinicians.

While evidence suggests that moral distress can contribute to burnout, many factors that contribute to burnout likely do so through other mechanisms.^44,45^ Here, we analyze data from a large study of US physicians that assessed both burnout (ie, EE and DP) and moral distress to explore the association between these constructs, the frequency with which they coexist, how often 1 condition may be present in the absence of the other, and their association with ITR and ITL. We also evaluated the prevalence of moral distress in physicians relative to other US workers.

## Methods

The sample for this study was assembled using the American Medical Association (AMA) Physician Professional Data (PPD), a nearly complete list of all US physicians regardless of AMA membership. Complete details of the 2023 survey methodology have been previously reported.^19^ The institutional review boards at Stanford University, University of Illinois Chicago, and Mayo Clinic reviewed and approved the study. The study followed the American Association for Public Opinion Research (AAPOR) reporting guideline for survey studies. Participation was voluntary and all responses were anonymous. Completion of the survey implied consent.

### Participants

#### Physician Survey and Population Sample

A sample of 90 000 physicians from all specialties was compiled from the AMA PPD and sent the online survey, with 6090 as undeliverable, resulting in a sample of 83 910. An additional sample of 8000 physicians received an incentivized online survey (ie, a $20 e-gift card for participation), with 551 undeliverable, resulting in a sample of 7449. Non–primary care physician specialties were oversampled to increase participation rates in less common specialties. Both samples were sent the survey link on October 19, 2023, followed by reminders over the ensuing weeks, and surveys completed by November 20, 2023, were included in the analysis.

A separate sample of 4000 physicians across all specialties was mailed a paper copy of the same survey on December 11, 2023, with 280 returned as undeliverable, resulting in a sample of 3720 physicians. A second copy of the survey was mailed on January 25, 2024, to nonresponders. All mailed surveys returned by March 5, 2024, were included in the analysis.

A secondary online survey of a random sample of 1000 physicians from the nonincentivized online survey who did not respond received an email invitation and were offered a $20 gift card on November 28, 2023, with 2 email reminders to participate in an abbreviated survey, resulting in 82 emails being undeliverable, leaving a sample of 918. Surveys completed by December 15, 2023, were included in the analysis. Participation in the online or mailed survey was voluntary, and responses were anonymous. As previously described, we also surveyed a probability-based sample of 3501 employed individuals aged 29 to 65 years from the general US population between November 21 and November 30, 2023, using Ipsos KnowledgePanel.^19^

### Study Measures

Both physicians and other US workers were queried on their demographics (eg, age, gender, race and ethnicity, relationship status, and hours worked per week were self-reported). Physicians were also asked about their specialty, years in practice, nights on call, and practice setting.

Moral distress was measured using the Moral Distress Thermometer (MDT),^46^ a frequently used tool^47,48,49,50,51,52^ that describes moral distress and asks responders to indicate the amount of moral distress related to work they experienced in the last 2 weeks. The 0 to 10 scale options included none, mild, uncomfortable, distressing, and worst possible (eFigure 1 in Supplement 1). A score of 4 or higher indicates high moral distress.^53^ Scores on the MDT have been shown to have moderate correlation with the longer 32-item Moral Distress Scale and to self-report ITL.^46^

Burnout in physicians was measured using the full-length EE and DP scales of the Maslach Burnout Inventory (MBI), used under license from Mind Garden, Inc.^33,54,55^ Consistent with the longstanding approach,^56,57,58^ physicians with high scores on the DP and/or EE scales were considered to have at least 1 manifestation of professional burnout. While the full-length scales were used in the physician study, single-item measures of EE and DP from the full MBI were used in the employed nonphysician US workers survey. These 2 items have demonstrated strong association with the full-length EE and DP scales, with the area under the ROC curve 0.94 and 0.93, respectively, in a sample of more than 10 000 individuals.^59,60^

Professional fulfillment was measured using the Stanford Professional Fulfillment Index (scale range: 0 to 10).^61^ ITL was measured with a standardized item that asked “what is the likelihood that you will leave your current practice within 2 years?” (answer choices: none, slight, moderate, likely, and definitely).^24,62,63^ ITR was measured by asking “what is the likelihood that you will reduce the number of hours you devote to clinical care over the next 12 months?” (answer choices: none, slight, moderate, likely, and definitely).^24,62,63^

### Statistical Analysis

Responses from the physician surveys were pooled for analysis. Respondents who were not in practice or retired were excluded. Standard descriptive statistics and McNemar tests were completed to identify the demographic characteristics of the physician and US nonphysician worker samples. Associations between moral distress and outcomes (EE, DP, professional fulfillment, ITL, ITR, and overall burnout) were examined using analysis of variance (ANOVA) for continuous variables and χ^2^ tests for categorical variables. Spearman correlation tested associations between moral distress scores and EE and DP scores. Multivariable logistic regression was used to explore associations between personal and professional characteristics and moral distress. Additionally, multivariable logistic regression adjusting for age, gender, relationship status, race, ethnicity, and weekly work hours, with and without education level, was used to examine differences in moral distress between US physicians and workers in other fields. All tests were 2-tailed with a significance level set at *P* < .05. Statistical analyses were performed in R version 4.5.0 (R Foundation for Statistical Computing). Data were analyzed from June 30 to October 20, 2025.

## Results

As previously reported,^19^ survey participants included 6288 of 83 910 physicians (7.5%) from the nonincentivized survey, 906 of 7449 physicians (12.2%) from the incentivized online survey, and 449 of 3720 physicians (12.1%) from the mailed paper survey for a total of 7643 responding physicians. The demographic characteristics of the pooled survey participants closely matched those of practicing US physicians, with a slightly higher proportion of women respondents (2587 of 6538 [39.6%] vs 354 775 of 935 096 [37.9%]; *P* < .001).^19^ No statistically significant differences were observed in EE, DP, or work-life integration scores between physicians who responded to the primary survey and those who responded only to the secondary (nonresponder) survey.

Among the 7643 responding physicians, 5741 (75.1%) who were still in practice (146 [1.9%] were excluded because they were retired or not in practice) completed the MDT. The median (IQR) age was 53 (44-62) years, and 3262 men (58.0%), 2255 women (40.1%), and 107 individuals who responded other (1.9%). Demographic characteristics of participants are shown in Table 1. Mean (SD) moral distress score was 3.29 (2.81) on the 0- to 10-scale, with 2243 of 5741 (39.1%) reporting a high level of moral distress (score of 4 or higher). On multivariable analysis controlling for personal characteristics (age, gender, relationship status, race, ethnicity) and professional characteristics (weekly work hours, number of nights on call per week, specialty, and practice setting), women had higher odds of high moral distress relative to men (OR, 1.29; 95% CI, 1.12-1.48) (Table 2). There were also differences in the odds of high moral distress by age with older physicians (age >65 years vs age <35 years: OR, 0.57; 95% CI, 0.39-0.84), and married physicians compared with single physicians (OR, 0.69; 95% CI, 0.56-0.84) had lower odds of high moral distress. Compared with internal medicine subspecialty physicians, emergency medicine physicians (OR, 3.16; 95% CI, 2.27-4.4) and general internal medicine physicians (OR, 1.92; 95% CI, 1.42-2.59) were at greater odds of having high levels of moral distress, while pathologists had lower odds of high levels of moral distress (OR, 0.43; 95% CI, 0.25-0.73). The odds of moral distress also increased with each additional hour worked per week (OR, 1.01; 95% CI, 1.01-1.02).

**Table 1.** Demographic Characteristics of Responding Physicians

Characteristics	Responders, No. (%) (n = 5741)^a^
Age, medan (IQR) y	53 (44-62)
<35	160 (3.1)
35-44	1240 (24.4)
45-54	1385 (27.2)
55-64	1395 (27.4)
≥65	910 (17.9)
Missing	651 (11.3)
Gender	
Man	3262 (58.0)
Woman	2255 (40.1)
Other	107 (1.9)
Missing	117 (2.0)
Race	
American Indian or Alaska Native	8 (0.2)
Asian	809 (15.8)
Black	168 (3.3)
Pacific Islander or Native Hawaiian	8 (0.2)
White	3799 (74.0)
More than 1 race	145 (2.8)
Other^b^	197 (3.8)
Missing	607 (10.6)
Ethnicity	
Hispanic or Latinx	335 (6.1)
Non-Hispanic	5143 (93.9)
Missing	263 (4.6)
Relationship status	
Single	638 (11.4)
Married	4594 (82.2)
Partnered	288 (5.2)
Widowed or widower	66 (1.2)
Missing	155 (2.7)
Age of youngest child, y	
<5	678 (12.2)
5-12	996 (17.8)
13-18	852 (15.3)
19-22	534 (9.6)
>22	1548 (27.7)
Missing	161 (2.8)
No children	972 (17.4)
Specialty	
Anesthesiology	261 (4.6)
Dermatology	135 (2.4)
Emergency medicine	340 (5.9)
Family medicine	394 (6.9)
General surgery	194 (3.4)
General surgery subspecialty	476 (8.3)
Internal medicine, general	467 (8.2)
Internal medicine subspecialty	588 (10.3)
Neurology	186 (3.2)
Neurosurgery	67 (1.2)
Obstetrics and gynecology	221 (3.9)
Ophthalmology	194 (3.4)
Orthopedic surgery	288 (5.0)
Otolaryngology	27 (0.5)
Other	368 (6.4)
Pathology	146 (2.5)
Pediatrics, general	298 (5.2)
Pediatric subspecialty	234 (4.1)
Physical medicine and rehabilitation	131 (2.3)
Prevention or occupational medicine	20 (0.3)
Psychiatry	430 (7.5)
Radiation oncology	39 (0.7)
Radiology	195 (3.4)
Urology	27 (0.5)
Missing	15 (0.3)
Years in practice, y	
<5	509 (8.9)
5-<10	776 (13.6)
10-<20	1522 (26.6)
20-<30	1452 (25.4)
≥30	1465 (25.6)
Missing	17 (0.3)
Hours worked per wk, h	
<40	1196 (21.0)
40-49	1310 (23.0)
50-59	1389 (24.3)
60-69	1149 (20.1)
70-79	296 (5.2)
≥80	367 (6.4)
Missing	34 (0.6)
Nights on call	
None	146 (2.6)
1	1774 (32.1)
≥2	3605 (65.2)
Missing	216 (3.8)
Primary practice setting	
Private practice	2915 (50.8)
Academic medical center	1801 (31.4)
Veterans’ hospital	151 (2.6)
Active military practice	32 (0.6)
Federally qualified health care center	166 (2.9)
Other	670 (11.7)
Missing	6 (0.1)

^a^ Percentages are calculated excluding missing data. Missing values’ percentages are calculated from total sample.

^b^ The Other race category included write-in responses that did not align with predefined or standard options.

**Table 2.** Association Between Demographic and Practice Characteristics and High Moral Distress

Outcome or estimator	OR (95% CI)	*P* value	Overall *P* value
**Moral distress (score ≥4)^a^**			
Age, y			
<35	1 [Reference]	NA	<.001
35-44	1.16 (0.81-1.66)	.43	
45-54	1.12 (0.78-1.61)	.55	
55-64	0.85 (0.59-1.22)	.37	
≥65	0.57 (0.39-0.84)	.01	
Gender			
Man	1 [Reference]	NA	<.001
Woman	1.29 (1.12-1.48)	<.001	
Other	1.72 (0.94-3.19)	.08	
Relationship status			
Single	1 [Reference]	NA	.002
Married	0.69 (0.56-0.84)	<.001	
Partnered	0.82 (0.59-1.14)	.23	
Widowed or widower	0.60 (0.31-1.14)	.13	
Race			
American Indian or Alaska Native	2.38 (0.49-12.60)	.27	.17
Asian	0.93 (0.78-1.10)	.40	
Black or African American	0.75 (0.51-1.08)	.13	
More than 1 race	0.75 (0.51-1.10)	.14	
Pacific Islander or Native Hawaiian	0.67 (0.09-3.18)	.63	
White	1 [Reference]	NA	
Other^b^	1.33 (0.91-1.93)	.14	
Ethnicity			
Hispanic or Latinx	1.14 (0.87-1.48)	.34	NA
Non-Hispanic and Latinx	1 [Reference]	NA	
Hours worked per wk (for each additional)	1.01 (1.01-1.02)	<.001	NA
Nights on call	1.03 (0.99-1.06)	.14	NA
Specialty			
Internal medicine subspecialty	1 [Reference]	NA	<.001
Anesthesiology	1.28 (0.89-1.83)	.19	
Dermatology	1.08 (0.67-1.71)	.76	
Emergency medicine	3.16 (2.27-4.40)	<.001	
Family medicine	1.33 (0.97-1.81)	.07	
General internal medicine	1.92 (1.42-2.59)	<.001	
General pediatrics	1.10 (0.77-1.56)	.61	
General surgery	1.09 (0.73-1.61)	.67	
General surgery subspecialty	1.11 (0.83-1.49)	.49	
Neurology	1.50 (0.99-2.27)	.06	
Neurosurgery	1.42 (0.78-2.57)	.25	
Obstetrics and gynecology	1.26 (0.87-1.83)	.22	
Ophthalmology	0.96 (0.62-1.46)	.84	
Orthopedic surgery	0.99 (0.69-1.41)	.94	
Other	1.22 (0.88-1.67)	.23	
Otolaryngology	0.78 (0.27-2.00)	.62	
Pathology	0.43 (0.25-0.73)	.002	
Pediatric subspecialty	1.22 (0.85-1.75)	.28	
Physical medicine and rehabilitation	1.43 (0.91-2.23)	.12	
Preventive or occupational medicine	2.18 (0.75-6.37)	.15	
Psychiatry	1.29 (0.95-1.75)	.11	
Radiation oncology	0.91 (0.41-1.93)	.81	
Radiology	0.97 (0.64-1.46)	.88	
Urology	0.32 (0.07-0.98)	.07	
Practice settings			
Private practice	1 [Reference]	NA	.003
Academic medical center	0.98 (0.84-1.14)	.80	
Veterans’ hospital	0.75 (0.49-1.13)	.17	
Federally qualified health care center	1.36 (0.93-1.99)	.11	
Active military practice	1.48 (0.63-3.44)	.36	
Other	1.43 (1.16-1.75)	.001	

Abbreviations: OR, odds ratio; NA, not applicable.

^a^ Core range 0 to 10; scores 4 or more considered high.^53^

^b^ The Other race category included write-in responses that did not align with predefined or standard options.

**Figure 1.**
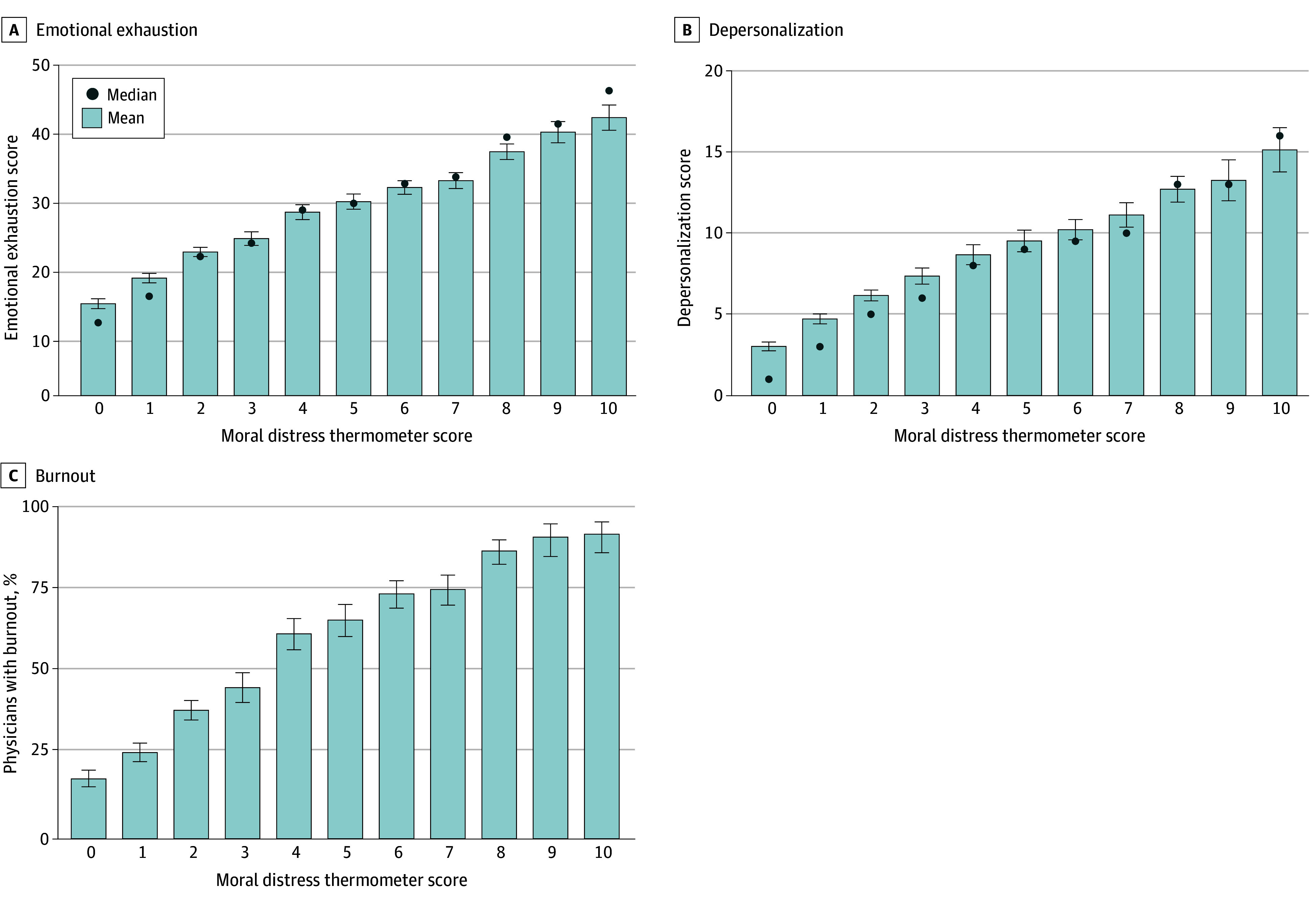
Bar Graph of the Association Between Moral Distress and Emotional Exhaustion, Depersonalization, and Burnout Among Physicians Score range between 0 and 10. Scores higher than 4 were considered high.^53^ Error bars indicate 95% CIs. Median burnout scores are not provided as the Maslach Burnout Inventory does not generate a composite burnout score; we report the proportion meeting clinical burnout criteria (high emotional exhaustion or depersonalization).

Moral distress and burnout exhibited a consistent association, with a burnout prevalence of 18.1% (169 of 932) and 92.4% (133 of 144) among physicians with a moral distress score of 0 and 10, respectively. Mean EE and DP score, as well as the proportion of physicians with burnout, were higher with each 1-point increase in moral distress score (Figure 1). The overall correlation between EE score and moral distress score was *R* = 0.55 (*P* < .001), while the correlation between DP score and moral distress score was *R* = 0.50 (*P* < .001). Overall, 1068 of 3477 physicians (30.7%) with a moral distress score of less than 4 had burnout compared with 1675 of 2231 physicians (75.1%) of those with scores of 4 or more (*P* < .001).

Among 5704 physicians with complete data, 3477 (61.0%) reported low moral distress (ie, a score of less than 4). However, even within this group, a substantial proportion experienced symptoms of burnout: 893 of 3477 (25.7%) had high EE, 613 of 3477 (17.6%) had high DP, and 1068 of 3477 (30.7%) had at least 1 burnout symptom. Conversely, among those with high EE or DP, nearly two-thirds also had high moral distress (ie, a score of 4 or more). Overall, among the 2739 physicians with 1 or more symptoms of burnout, 1671 (61.0%) had a moral distress score 4 or more. A Venn diagram showing the proportions of physicians with high EE, high DP, and high moral distress is shown in eFigure 2 in Supplement 1.

**Figure 2.**
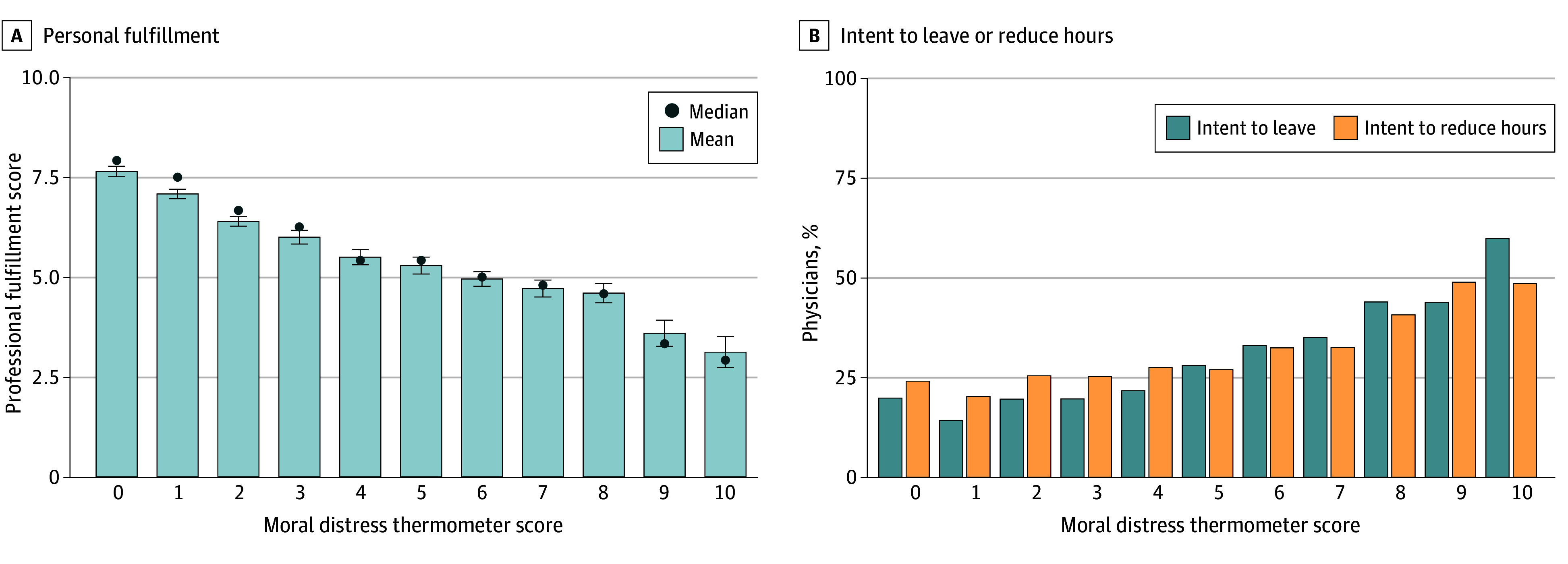
Bar Graph of Association Between Moral Distress and Professional Fulfillment Among Physicians and Proportion of Physicians Across Moral Distress Scores Who Intend to Reduce Hours or Leave Practice Error bars indicate 95% CIs.

Mean professional fulfillment score was lower for each 1-point increase in moral distress score (Figure 2A). Physicians with low moral distress (ie, a score less than 4) were substantially more likely to report high professional fulfillment (1617 of 3474 [46.5%]) compared with those with high moral distress (284 of 2222 [12.8%]) (*P* < .001). ITL and ITR increased with each point increase on the moral distress scale (Figure 2B). Specifically, 619 of 3404 physicians (18.2%) with low moral distress reported ITL within 24 months compared with 748 of 2171 physicians (34.4%) with high moral distress (*P* < .001). Similarly, 807 of 3416 physicians (23.6%) with low moral distress intended to reduce clinical hours in the next year compared with 739 of 2177 (33.9%) with high moral distress (*P* < .001).

Finally, we compared moral distress scores among employed physicians and US workers in other fields. The demographic characteristics of physicians and the general working population are shown in Table 3. The median (IQR) moral distress score was higher for physicians than workers in other fields (3 [1-6] vs 0 [0-2]; *P* < .001). In aggregate, 1796 of 4322 (41.6%) physicians and 406 of 2864 (14.2%) of US workers in other fields reported a high level of moral distress. More than half of US workers (1581 of 2864 [55.2%]) had an MDT score of 0, and the distribution of the MDT scores is more right skewed compared with physicians (eFigure 3 in Supplement 1). After adjusting for age, gender, relationship status, and hours worked per week, physicians remained at greater odds of having high moral distress than US workers in other fields (OR, 4.40; 95% CI, 3.84-5.06) (eTable 1 in Supplement 1). After the addition of level of education to the multivariable model (eTable 2 in Supplement 1), physicians had greater odds of moral distress than all other levels of education (professional or doctoral degree other than medical doctor or doctor of osteopathic medicine: OR, 0.11 [95% CI, 0.06-0.19]; master’s degree or higher: OR, 0.25 [95% CI, 0.19-0.32]; Bachelor’s degree: OR, 0.21 [95% CI, 0.17-0.27]; some college or associate degree: OR, 0.23 [95% CI, 0.18-0.28]; high school graduate: OR, 0.25 [95% CI, 0.20-0.32]; less than high school graduate: OR, 0.38 [95% CI, 0.22-0.62]).

**Table 3.** Comparison of Physicians in the Sample Aged 29 to 64 Years With a Probability-Based Sample of the Working US Population Aged 29 to 64 Years in 2023

Characteristic	Participant, No. (%)		*P* value
	Physicians (n = 4890)^a^	Population (n = 2867)^b^	
Gender or sex^c^			
Men	2653 (55.2)	1596 (55.7)	.70
Women	2154 (44.8)	1271 (44.3)	
Missing^d^	83 (1.7)	0	
Age, y			
Median (IQR)	50.0 (42.0-58.0)	50.0 (41.0-57.0)	.03
29-34	187 (3.8)	239 (8.3)	<.001
35-44	1439 (29.4)	733 (25.6)	
45-54	1557 (31.8)	925 (32.3)	
55-64	1707 (34.9)	970 (33.8)	
Missing^d^	0	0	
Race			
American Indian or Alaska Native	7 (0.2)	19 (0.7)	<.001
Asian	806 (17.8)	148 (5.2)	
Black	155 (3.4)	305 (10.6)	
More than 1 race	135 (3.0)	117 (4.1)	
Pacific Islander or Native Hawaiian	8 (0.2)	1 (0.0)	
White	3232 (71.4)	2277 (79.4)	
Other^e^	183 (4.0)	0	
Missing^d^	364 (7.4)	0	
Ethnicity			
Hispanic/Latinx	322 (6.7)	382 (13.3)	<.001
Non-Hispanic	4453 (93.3)	2485 (86.7)	
Missing^d^	115 (2.4)	0	
Relationship status			
Single	564 (11.6)	779 (27.2)	<.001
Married	3998 (82.2)	1863 (65.0)	
Partnered	266(5.5)	171 (6.0)	
Widowed or widower	34 (0.7)	54 (1.9)	
Missing^d^	294 (6.0)	0	
Hours worked per wk, h			
Median	50.0 (40.0-60.0)	40.0 (40.0-45.0)	<.001
<40	868 (17.8)	634 (22.2)	<.001
40-49	1206 (24.8)	1709 (59.8)	
50-59	1241 (25.5)	363 (12.7)	
60-69	979 (20.1)	117 (4.1)	
70-79	243 (5.0)	14 (0.5)	
≥80	327 (6.7)	23 (0.8)	
Missing^d^	26 (0.5)	7 (0.2)	
Highest level of education completed			
Less than high school graduate	NA	83 (2.9)	NA
High school graduate	NA	571 (19.9)	
Some college or associate degree	NA	774 (27.0)	
Bachelor’s degree	NA	802 (28.0)	
Master’s degree or higher	NA	484 (16.9)	
Professional or doctorate degree (other than MD/DO)	NA	153 (5.3)	
MD/DO	4890 (100.0)	NA	
Occupation			
Professional^f^	NA	1492 (52.1)	NA
Health care^g^	NA	104 (3.6)	
Service^h^	NA	215 (7.5)	
Sales^i^	NA	171 (6.0)	
Office and administrative support	NA	264 (9.2)	
Farming, forestry fishing	NA	14 (0.5)	
Precision production, craft and repair^j^	NA	183 (6.4)	
Transportation and material moving	NA	103 (3.6)	
Armed services	NA	9 (0.3)	
Other	NA	311 (10.9)	
Missing^d^	NA	1 (0)	
Moral distress			
Median (IQR)	3.0 (1.0-6.0)	0 (0-2.0)	<.001
Missing^d^	568 (11.6)	3 (0.1)	

Abbreviations: DO, doctor of osteopathic medicine; MD, medical doctor; NA, not applicable.

^a^ Physician data includes responders to the mailed and online survey age 29 to 65 years and actively employed at the time of the survey as well as responders to the secondary (nonresponder) survey meeting these criteria.

^b^ Age 29 to 65 years and actively employed at the time of the survey.

^c^ Participants in the physician survey provided information on gender (eg, man, woman, nonbinary, another gender, prefer not to say) while participants in the population survey provided information on sex (response options: male, female, other).

^d^ Percentages are calculated excluding missing data. Missing values’ percentages are calculated from total sample.

^e^ The Other race category included write-in responses that did not align with predefined or standard options.

^f^ Business or financial; management; computer or mathematical; architecture or engineering; lawyer or judge; life, physical, or social sciences; community or social services; teacher nonuniversity; teacher college or university; other.

^g^ Nurse, pharmacist, paramedic, lab technician, nursing aide, orderly, dental assistant.

^h^ Protective service, food preparation or service, building cleaning or maintenance, personal care or service.

^i^ Sales representative, retail sales, other sales.

^j^ Construction and extraction; installation, maintenance, or repair; precision production (eg, machinist, welder, backer, printer, tailor).

## Discussion

The findings from this large national survey study suggest high levels of moral distress are common among US physicians. In aggregate, approximately 2 of every 5 physicians reported experiencing a high level of moral distress associated with work in the last 2 weeks. After adjusting for age, gender, relationship status, hours worked per week, and education, physicians remained at higher odds for moral distress compared with US workers in other fields. Women physicians and those working more hours per week had higher odds of moral distress, while those who were older or married had lower odds.

Moral distress was associated with multiple undesirable conditions, including higher levels of burnout, lower levels of professional fulfillment, and increased odds of ITL and ITR. In addition, it has been previously reported that moral distress can lead to anger, frustration, depression, and guilt, aligning with an overall sense of powerlessness within physicians.^3^ In a study of intensive care unit clinicians, the most common emotion associated with moral distress was frustration.^64^ In a study of health care workers, the type of moral injury (ie, witnessing vs participating in a potentially morally injurious event) was associated with the consequences, with witnessing an event more likely to lead to turnover, whereas participating in an event was more likely to lead to burnout.^45^

Data from this large survey of US physicians demonstrates that, while work-related moral distress and burnout are correlated and frequently co-occur, they remain distinct constructs. In this sample, based on the correlation coefficients, moral distress accounted for 30% of the variability in EE and 25% of the variability in DP, and vice versa. Notably, 3 in 4 physicians with high moral distress exhibited at least 1 symptom of burnout compared with less than 1 in 3 of those with low moral distress.

Recognizing the distinction between moral distress and burnout is essential for developing targeted organizational interventions. While some interventions may address both moral distress and burnout, other interventions may target one more effectively than the other. Addressing structural barriers, such as those created by employers, health systems, or payers, that prevent physicians from acting in accordance with their ethical values may help mitigate moral distress and indirectly improve burnout.^44^ For example, organizations can prioritize ethics as a core value, empowering physicians to act in patients’ best interests rather than prioritizing financial outcomes.^44^ Simultaneously, workplace factors that contribute to EE and DP, such as suboptimal teamwork, inadequate flexibility or control over work, and excessive documentation burden, should be addressed to mitigate burnout and may indirectly alleviate moral distress. Consistently, perceived values misalignment between health care professionals’ values and those of their institution has been shown to contribute to both burnout and moral distress.^65^

Organizational efforts to reduce moral distress should prioritize open communication and systematic approaches that align patient care, especially around patient preferences and team consensus, helping to avoid care that is perceived as futile.^66^ Creating dedicated spaces for care teams to discuss ethical challenges fosters a sense of institutional support and shared understanding.^3^ Peer support programs to build moral resilience, access to consultation for ethical dilemmas, and investment in mental health are also likely to mitigate moral distress.^67^ Additionally, empowering physicians with greater agency and control over their work can further reduce moral distress.^2,68^ Encouraging the recognition and normalization of conflicting emotions supports self-awareness and promotes a thoughtful, professional response to challenging situations.^69^

### Limitations

This study has several limitations, including potential response bias due to a low survey participation rate, a common challenge in large national survey studies.^70,71,72,73,74^ Although this raises questions about the representativeness of participants and generalizability, respondents were generally similar to the broader US physician population with respect to age and gender.^19^ A secondary survey of nonresponders found no significant differences in burnout and satisfaction with work-life integration, supporting the representativeness of the sample.^19^ The self-report study design may also be susceptible to social desirability bias. Given the cross-sectional nature of the study, we are unable to determine the causal relationship between variables or the potential direction of effect. Additionally, while moral distress was measured using the widely adopted MDT, this single-item measure does not capture the full complexity of moral distress compared with a longer, multi-item assessment.^46^ Future research on the types and impacts of various interventions on moral distress, and their association with burnout, would be useful.

## Conclusions

Nearly 2 in 5 physicians reported experiencing a high level of moral distress associated with their work in the last 2 weeks. Physicians are at a higher risk of moral distress than the general US working population. Moral distress in physicians is associated with multiple unfavorable outcomes, including occupational burnout, ITL, and ITR. Occupational burnout and moral distress are distinct forms of work-related distress that often coexist and may contribute to one another. Interventions are needed at both the organization and system level to address the underlying factors that contribute to moral distress and occupational burnout.

## Notes

Supplement 1. **eFigure 1.** Moral distress thermometer **eFigure 2.** Relationships between high moral distress, high emotional exhaustion and high depersonalization among physicians with one or more conditions (N=3295) **eFigure 3.** Prevalence of MDT scores by cohort **eTable 1. **Pooled analysis of physicians and US workers age 29 to 64 years to identify factors associated with moral distress **eTable 2. **Pooled analysis of physicians and US workers age 29 to 64 years to identify factors associated with moral distress after adjusting for level of education.

Supplement 2.
Data Sharing Statement
