# Supplementary material for: Moral Distress and Occupational Burnout in US Physicians
Source: JAMA Netw Open. 2026 Mar 24;9(3):e263161. doi: 10.1001/jamanetworkopen.2026.3161 (PMC13014174; doi:10.1001/jamanetworkopen.2026.3161)
Supplement: Supplement 1. — eFigure 1. Moral distress thermometer eFigure 2. Relationships between high moral distress, high emotional exhaustion and high depersonalization among physicians with one or more conditions (N=3295) eFigure 3. Prevalence of MDT scores by cohort eTable 1. Pooled analysis of physicians and US workers age 29 to 64 years to identify factors associated with moral distress eTable 2. Pooled analysis of physicians and US workers age 29 to 64 years to identify factors associated with moral distress after adjusting for level of education. [file jamanetwopen-e263161-s001.pdf]

Supplemental Online Content

Tutty MA, West CP, Dyrbye LN, et al. Moral distress and occupational burnout in US physicians. *JAMA Netw Open*. 2026;9(3):e263161.
doi:10.1001/jamanetworkopen.2026.3161

eFigure 1. Moral distress thermometer
eFigure 2. Relationships between high moral distress, high emotional exhaustion and high depersonalization among physicians with one or more conditions (N=3295)
eFigure 3. Prevalence of MDT scores by cohort
eTable 1. Pooled analysis of physicians and US workers age 29 to 64 years to identify factors associated with moral distress
eTable 2. Pooled analysis of physicians and US workers age 29 to 64 years to identify factors associated with moral distress after adjusting for level of education

This supplemental material has been provided by the authors to give readers additional information about their work.

© 2026 Tutty MA et al. *JAMA Network Open*.

eFigure 1: Moral distress thermometer

Moral distress is a form of distress that occurs when you believe you know the ethically correct thing to do, but something or someone restricts your ability to pursue the right course of action. Please circle the number (0–10) on the thermometer that best describes how much moral distress you have been experiencing related to work in the past 2 weeks, including today.

None		Mild		Uncomfortable		Distressing		Intense		Worst Possible
0	1	2	3	4	5	6	7	8	9	10

© 2026 Tutty MA et al. *JAMA Network Open*.

eFigure 2: Relationships between high moral distress, high emotional exhaustion and high depersonalization among physicians with one or more conditions (N=3295)

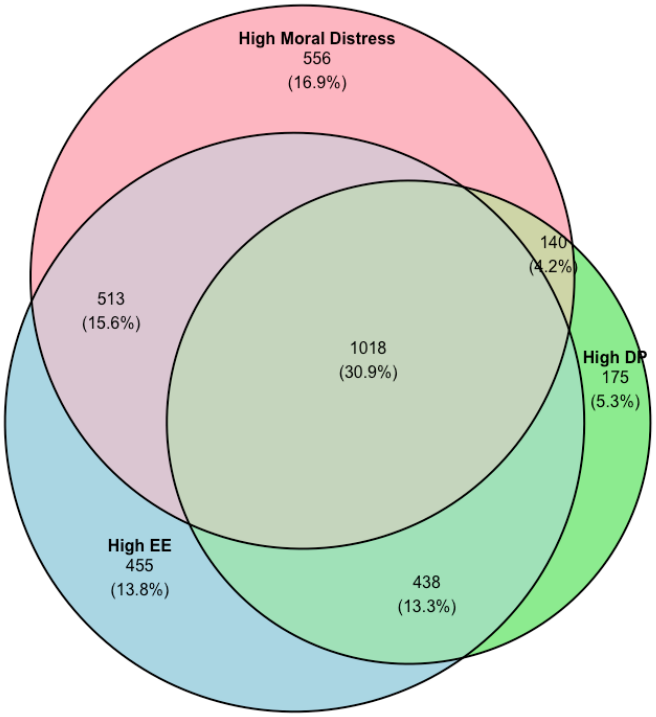

© 2026 Tutty MA et al. *JAMA Network Open*.

eFigure 3. Prevalence of MDT scores by cohort

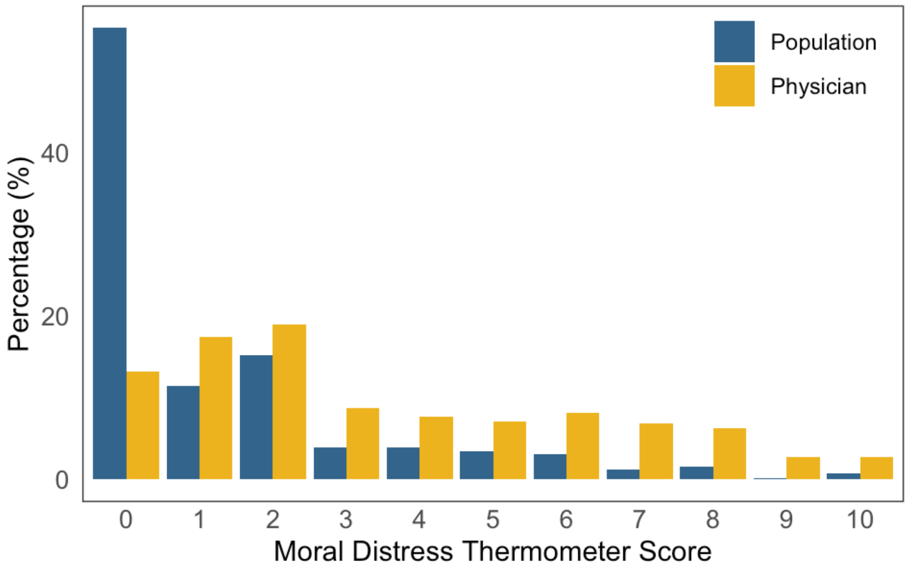

© 2026 Tutty MA et al. *JAMA Network Open*.

eTable 1: Pooled analysis of physicians and US workers age 29-64 years to identify factors associated with moral distress

Outcome	Predictor	OR (95% CI)	P-value	Overall P-value
Moral Distress	Age (vs. age <35)			<.001
	35-44	1.03 (0.80-1.34)	0.83	
	45-54	0.88 (0.68-1.14)	0.33	
	55-64	0.67 (0.52-0.87)	0.002	
	Gender(vs. man)			<.001
	Woman	1.25 (1.11-1.40)	<0.001	
	Other	1.77 (0.96-3.29)	0.07	
	Relationship status (vs. single)			.002
	Married	0.75 (0.65-0.88)	<0.001	
	Partnered	0.96 (0.74-1.25)	0.76	
	Widowed/widower	0.85 (0.47-1.49)	0.59	
	Race (vs White)			.20
	American Indian or Alaska Native	1.46 (0.54-3.57)	0.43	
	Asian	0.95 (0.81-1.12)	0.54	
	Black	1.06 (0.82-1.35)	0.65	
	More than 1 race	0.72 (0.52-0.99)	0.047	
	Pacific Islander or Native Hawaiian	0.57 (0.08-2.59)	0.50	
	Other [a]	1.34 (0.93-1.93)	0.12	
	Ethnicity (Hispanic or Latinx vs. non-Hispanic and Latinx)	1.40 (1.15-1.70)	0.001	
	Hours worked per week (each additional hour)	1.01 (1.01-1.01)	<0.001	
	Physician vs. other US workers	4.40 (3.84-5.06)	<0.001	

[a] The ‘Other’ race category included write-in responses that did not align with predefined or standard options.

© 2026 Tutty MA et al. *JAMA Network Open*.

eTable 2: Pooled analysis of physicians and US workers age 29 to 64 years to identify factors associated with moral distress after adjusting for level of education

Outcome	Predictor	OR (95% CI)	P-value	Overall P-value
Moral Distress	Age (vs. age <35)			<.001
	35-44	1.01 (0.78-1.32)	0.91	
	45-54	0.87 (0.67-1.13)	0.29	
	55-64	0.66 (0.51-0.86)	0.002	
	Gender(vs. man)			<.001
	Woman	1.24 (1.11-1.39)	<0.001	
	Other	1.78 (0.97-3.30)	0.70	
	Relationship status (vs. single)			.003
	Married	0.76 (0.65-0.89)	0.001	
	Partnered	0.96 (0.74-1.25)	0.76	
	Widowed/widower	0.84 (0.46-1.47)	0.56	
	Race (vs White)			.22
	American Indian or Alaska Native	1.33 (0.49-3.26)	0.55	
	Asian	0.95 (0.81-1.12)	0.56	
	Black	1.06 (0.82-1.35)	0.67	
	More than 1 race	0.72 (0.52-0.99)	0.049	
	Pacific Islander or Native Hawaiian	0.57 (0.08-2.59)	0.50	
	Other [a]	1.34 (0.93-1.94)	0.11	
	Ethnicity (Hispanic or Latinx vs. non-Hispanic and Latinx)	1.36 (1.11-1.65)	0.002	
	Hours worked per week (each additional hour)	1.01 (1.01-1.01)	<0.001	
	Highest Level of Education Completed (vs. physicians)			<.001
	Less than high school graduate	0.38 (0.22-0.62)	<0.001	
	High school graduates	0.25 (0.20-0.32)	<0.001	
	Some college or associate degree	0.23 (0.18-0.28)	<0.001	
	Bachelor’s degree	0.21 (0.17-0.27)	<0.001	
	Master’s degree or higher	0.25 (0.19-0.32)	<0.001	
	Professional or doctorate degree (other than MD/DO)	0.11 (0.06-0.19)	<0.001	

[a] The ‘Other’ race category included write-in responses that did not align with predefined or standard options.

© 2026 Tutty MA et al. *JAMA Network Open*.
