# Supplementary material for: Moral Distress and Occupational Burnout in US Physicians
Source: JAMA Netw Open. 2026 Mar 24;9(3):e263161. doi: 10.1001/jamanetworkopen.2026.3161 (PMC13014174; doi:10.1001/jamanetworkopen.2026.3161)
Supplement: Supplement 2. — Data Sharing Statement [file jamanetwopen-e263161-s002.pdf]

Data Sharing Statement

Tutty. Moral Distress and Occupational Burnout in US Physicians. *JAMA Netw Open*.
Published March 24, 2026. doi:10.1001/jamanetworkopen.2026.3161

Data
Data available: No

Additional Information
Explanation for why data not available: Data are still being used for other protocol specified analysis.
